# Alexithymia is associated with insomnia in Chinese patients with schizophrenia

**DOI:** 10.3389/fpsyt.2023.1252763

**Published:** 2023-12-15

**Authors:** Fangfang Cai, Huixia Jiang, Siyu Tong, Siyao Zhou, Mengpu Wang, Shiyu Sun, Jie Liu, Yao Xu, Nankai Lin, Jiajing Dai, Xinyao Wang, Wei Wang, Ke Zhao, Xixi Wu

**Affiliations:** ^1^School of Mental Health, Wenzhou Medical University, Wenzhou, China; ^2^Renji College of Wenzhou Medical University, Wenzhou, China; ^3^The Affiliated Kangning Hospital, Wenzhou Medical University, Wenzhou, China; ^4^Lishui Second People’s Hospital Affiliated to Wenzhou Medical University, Lishui, China; ^5^The Affiliated Kangning Hospital of Wenzhou Medical University, Zhejiang Provincial Clinical Research Center for Mental Disorder, Wenzhou, China; ^6^Wenzhou Lucheng District Third People’s Hospital, Wenzhou, China

**Keywords:** alexithymia, insomnia, schizophrenia, negative symptom, recognition

## Abstract

**Background:**

Sleep disorders are prevalent among patients with schizophrenia and are associated with several negative consequences. Although, researchers have recently suggested that sleep disorders have a close correlation with alexithymia, and schizophrenia also has a strong correlation with alexithymia, there have been few studies on the relationships between schizophrenia, sleep disorders and alexithymia. Therefore, this study aimed to explore the relationships between psychiatric symptoms, alexithymia and sleep problems in patients with schizophrenia so as to provide a reference for the clinical treatment of this comorbidity.

**Methods:**

In total, 977 patients with schizophrenia were recruited for this study. The Insomnia Severity Index (ISI) was used to assess sleep disorders, and the Positive and Negative Syndrome Scale (PANSS), Repeatable Battery for the Assessment of Neuropsychological Status (RBANS) and Toronto Alexithymia Scale (TAS) were used to evaluate clinical symptoms, cognitive functions and the ability to express emotion, respectively.

**Results:**

The results indicated that the PANSS subscales (G-subscore) and TAS group were risk factors for insomnia in schizophrenia patients (all *p* < 0.05). The mediation model showed the standardized path coefficients from schizophrenia to alexithymia (*β* = 0.104, *p* < 0.001) and from alexithymia to insomnia (*β* = 0.038, *p* < 0.001) were statistically significant.

**Conclusion:**

The results of this study indicated that alexithymia is associated with sleep disturbance in patients with schizophrenia. These findings may provide a new avenue for the treatment of schizophrenia patients with sleep disorders.

## 1 Introduction

Schizophrenia (SCZ) is a complex, severe mental illness characterized by psychotic symptoms such as hallucinations and delusions, negative symptoms such as affective flattening and poverty of thought, and cognitive impairments including attentional deficits and allomnesia (1, 2). While not considered a hallmark symptom, disordered sleep is also common among individuals with SCZ (3, 4). Research suggests that up to 30% of patients with SCZ have comorbid insomnia (5, 6). The most obvious manifestations of sleep abnormalities among patients with SCZ include increased sleep latency, reduced total sleep time and reduced sleep efficiency (7). These sleep disturbances often lead to poor clinical outcomes (8, 9), including more severe illness (10). For example, it has been reported that sleep issues can exacerbate paranoia and hallucinatory experiences (11), while improved sleep can reverse this phenomenon (12, 13). Moreover, several studies have reported that sleep deprivation may aggravate cognitive impairments and improved sleep quality might lead to better cognitive function (14, 15). Sleep disturbances may also be a risk factor for suicide attempts among patients with SCZ spectrum disorders (16). Given the harm brought about by insomnia in SCZ patients, the current study focused on investigating the potential mechanism behind the sleep disturbances observed in SCZ.

Alexithymia was originally proposed by Nemiah and Sifneos (17), to describe the cognitive and emotional deficits of psychosomatic patients in identifying, distinguishing, and expressing their emotions. Since then, this term has been defined as one type of cognition disorders, including having main difficulties in distinguishing and describing emotions (18–20). More and more studies have linked schizophrenia to alexithymia (21–23). For instance, Heshmati et al. found that alexithymia level in patients with schizophrenia spectrum disorders was further than patients with non-psychotic disorders and normal group (22). Another study investigated 60 individuals with psychiatric problems and found that those with a lifetime history of psychotic symptoms had higher alexithymia (24).

A series of studies have further found the presence of alexithymia among persons with schizophrenia link to other symptoms of illness. For instance, Fogley et al. (25) found neurocognitive impairments were related to difficulty identifying feelings and overall level of alexithymia, although they did not find significant links between alexithymia and positive or negative symptoms. In addition, Yi et al. (26) revealed that education levels, negative symptoms, and depressive symptoms were independently associated with alexithymia by multivariate regression analysis. Besides, they discovered the proportion of alexithymia was relative higher in those with stable conditions. What is more, Van’t Wout et al. (27) suggested that male patients with schizophrenia were more likely to experience alexithymia. The relationship between symptoms of schizophrenia and alexithymia is currently unclear, which poses great challenges and opportunities for related research.

There are not many articles on the pathological mechanism of alexithymia in schizophrenia. Studies have pointed out that patients with schizophrenia prone to having abnormalities in the key sections of the brain that is responsible for emotional processing, leading to reduced or diminished expression of emotions; this may make it difficult for these individuals to process emotions, which result in alexithymia (25, 27). Similarly, Kubota et al. (28, 29) noted that schizophrenia might cause impaired self-emotional awareness by damaging the integrity of white matter, or cause deficits in self-other distinction, self-disturbance, and language processing by disrupting left supramarginal gyrus, and ultimately lead to alexithymia. In brief, more research is expected to give to uncover the mysterious veil between schizophrenia and alexithymia.

Alexithymia is not only related to schizophrenia, but also related to sleep disorders (30). For example, one study reported that insomnia-related symptoms were associated with alexithymic features and depressive symptoms (31), while another study reported that alexithymic patients more commonly experienced severe depression and sleep disturbances than non-alexithymic patients (32). More than that, several studies have showed that higher levels of alexithymia may bring about sleep problems, such as insomnia, somnolence, nightmares, and somnambulism (30, 33, 34). These abnormalities of sleep commonly involve rapid eye movement (REM) alterations and impairments of dreaming process. REM sleep and dreaming have an important role in the emotional regulation and emotional memory consolidation (35, 36). If the normal dreaming process is interrupted, also known as nightmares, it will exhibit a failure to regulate and integrate emotions (33). And having alexithymic personality style may be one of the potential causes of nightmares (37). In a word, alexithymia is a result of impaired emotional regulation in the brain (25, 27), which may lead to abnormal dreaming processes or REM interruption, ultimately affecting sleep.

Many clinical and basic studies have revealed links between sleep disturbance, alexithymia and mental illness. For instance, a cross-sectional study found that alexithymia may be a potential additive to depression and one of the factors that can perpetuate insomnia (31). Another study found that alexithymia was associated with an increased prevalence of chronic sleep deprivation, depression, burnout and reduced empathy among a cohort of medical interns (38). However, the potential relationships between sleep deprivation, alexithymia and SCZ have received less attention, with only one published study to date. This cross-sectional study of 2,626 college students found a positive correlation between schizotypal traits and insomnia severity levels among college students, with alexithymia acting as a potential mediator of this relationship (39). However, the sample of the above study was freshman-year medical students rather than patients diagnosed with SCZ (39). Therefore, whether there exists a link between sleep disorders and alexithymia in patients with SCZ remains unknown.

The current study also investigates whether alexithymia may be a potential mediator in the relationship between schizophrenia and sleep disturbance. Because several articles have found that alexithymia is a mediator between sleep disorders and other psychological and psychiatric diseases. For example, Rehman et al. (40) found that alexithymia potentially mediates the relationship between sleep and paranoia as an important emotion mediator. Similarly, Pei et al. (41) noted that alexithymia might play a chain mediation role from childhood trauma to sleep problems in adolescents with depression. Furthermore, as mentioned above that alexithymia is, respectively, associated with schizophrenia and sleep disorders, it is reasonable to hypothesis that alexithymia may be a potential mediator between them.

Based on the literature described above, it is reasonable to speculate that there may be an association between sleep disturbances and alexithymia in patients with SCZ. Thus, the current study explored factors related to sleep disturbances among patients with chronic SCZ in China. It was hypothesized that SCZ patients with alexithymia would be more likely to report insomnia than those without alexithymia.

## 2 Materials and methods

### 2.1 Participants

**Figure 1:**
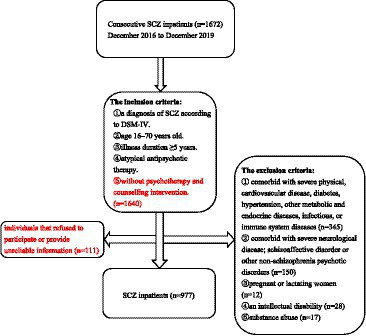
Sample flow chart.

A total of 977 consecutive inpatients with SCZ were recruited from the Affiliated Kangning Hospital of Wenzhou Medical University from December 2016 to December 2019. The inclusion criteria were: (1) age 16–70 years, Han nationality; (2) confirmation of a diagnosis of SCZ by two experienced psychiatrists based on the Structured Clinical Interview for DSM-IV (SCID); (3) illness duration of at least five years and being in the maintenance treatment with antipsychotic drugs to reduces the risk of relapse, which is recommended by guidelines (42, 43) to those have been after successful treatment of an acute episode; (4) the mean antipsychotic dose is approximately 30% lower than the dose used during acute phase according to clinical doctor’s advice and do not have a relapse of symptoms at that dose; and (5) without psychotherapy and counseling intervention. The exclusion criteria were: (1) comorbid severe physical diseases, cardiovascular diseases, diabetes, hypertension, other metabolic and endocrine diseases, infectious or immune system diseases; (2) comorbid severe neurological diseases, schizoaffective disorder or other non-SCZ psychotic disorders; (3) pregnant or lactating women; (4) intellectual disability; and (5) substance abuse. The participant details are presented in Figure 1.

Considering the wide age range, we divided the participants into three groups in accordance with World Health Organization (WHO) criteria: group I, young people (age < 44 years); group II, middle age (age 45–59 years) and group III, elderly (age 60 years or more) (44).

This study was approved by the Ethics Committee of the Affiliated Kangning Hospital of Wenzhou Medical University. All participants provided written informed consent before formal participation. This study was performed in accordance with the Declaration of Helsinki.

### 2.2 Clinical assessments

The Toronto Alexithymia Scale-20 (TAS-20) was used to measure three dimensions of alexithymia, namely Difficulties Identifying Feelings (TAS-DIF) (Factor 1), Difficulties Describing Feelings (TAS-DDF) (Factor 2) and Externally Oriented Thinking (TAS-EOT) (Factor 3) (45, 46). Due to its high internal consistency, stability and replicability, the TAS-20 has been widely used to comprehensively evaluate the presence and severity of alexithymia (47). Each item is rated on a five-point scale, ranging from 1 (“strongly disagree”) to 5 (“strongly agree”). Thus, the total score ranges from 20 to 100. Generally, a higher TAS score reflects more severe alexithymia. The Cronbach’s alpha coefficient of this scale was 0.82.

The Positive and Negative Syndrome Scale (PANSS) was used to evaluate patients’ psychotic symptoms (48). The PANSS consists of 30 items and three supplementary items, each of which is scored on a seven-point scale according to the severity of the symptom being assessed (48). The PANSS is a combination of the Concise Psychiatric Scale and the Psychopathological Rating Scale, that can be used to distinguish different types of SCZ and assess the severity of symptoms. The Cronbach’s alpha coefficient of this scale was 0.84.

The Repeatable Battery for the Assessment of Neuropsychological Status (RBANS) was used to evaluate cognitive deficits and motivational problems in patients with schizophrenia spectrum disorders (49). The RBANS contains 12 items or skills (List Learning, Story Memory, Figure Copy, Line Orientation, Picture Naming, Semantic Fluency, Digit Span, Coding, List Recall, List Recognition, Story Recall, and Figure Recall) and these items are combined to measures five cognitive domains, including immediate memory, visuospatial structure, language, attention and delayed memory (50, 51). It has good sensitivity, reliability and validity, and is widely used in SCZ populations (50). The Cronbach’s alpha coefficient was 0.87.

The Insomnia Severity Index (ISI) consists of seven items that are used to assess the severity of insomnia symptoms (52). The Cronbach’s alpha of the ISI is 0.85, indicating that it has good internal consistency. Scores for each item range from 0 to 4, with higher scores indicating more severe insomnia. The total ISI score ranges from 0 to 28. A total score of 0–7 represents no clinically significant insomnia; scores of 8–14 represent subclinical insomnia; scores of 15–21 represent moderate insomnia; and scores of 22–28 represent severe clinical insomnia. In this study, the ISI total score was modelled as both a continuous and categorical (0–7 or > 7) variable. Individuals with scores ranging from 0 to 7 were categorised as the no insomnia group, while those with scores >7 comprised the insomnia group (53). In this study, subjects’ subjective evaluations of sleep quality constituted the main source of data. As a self-rating scale, the ISI is a convenient tool for screening insomnia and is also useful for comparing the nature and symptoms of sleep disorders at different stages of treatment or between different people (54).

Split – half coefficient and internal concordance coefficient (Cronbach’s alpha coefficient) were utilized to test the reliability of each questionnaire. Coefficient of all questionnaires ranged from 0.82 to 0.87, which indicated that all questionnaires have good internal concordance and high split – half reliability.

### 2.3 Statistical analyses

*T*-tests and the χ2 test were used to identify differences between the SCZ patients with insomnia and those without insomnia. Furthermore, a binary logistic stepwise regression model was employed to analyze the risk factors for insomnia among patients with SCZ. Based on the correlation analysis, the PANSS subscales (P-subscore, N-subscore, G-subscore), RBANS subscales (Attention, Delayed memory, Language) and TAS-20 subscales (TAS-DIF, TAS-DDF) were included as variables in the regression model. Further, Pearson correlation analysis was performed to examine the correlations between the TAS, PANSS and RBANS in SCZ patients with insomnia. The PROCESS macro for SPSS was adopted to analyze the mediation effect of alexithymia on the relationship between schizophrenia and insomnia (55). The PROCESS macro for SPSS was adopted to analyze the mediation effect of alexithymia on the relationship between schizophrenia and insomnia (55). In the analysis, we used the Bootstrap method proposed by Preacher and Hayes (56) for mediating effect testing (Model 4) to test the mediating effect and calculated the bias corrected 95% confidence interval (CI) with 5,000 bootstrapping resamples. All statistical analyses were performed using IBM SPSS Statistics 25. The threshold for statistical significance was *p* < 0.05.

## 3 Results

A total of 977 patients with SCZ were recruited for this study. The patients were divided into two groups, SCZ with alexithymia group (*n* = 320) and SCZ without alexithymia group (*n* = 657), based on recommended cut-off point (TAS-20 ≥ 61) (25, 57). Table 1 showed that the two groups did not differ significantly with respect to age, P-subscore and Immediate memory (all *p* > 0.05), while Gender and Education were related to alexithymia in the SCZ. Besides, SCZ patients with alexithymia exhibited higher scores on the PANSS scale (total score (T-score), N-subscore and G-subscore) and lower scores on the RBANS (Total score, Attention, Visuospatial, Delayed Memory and Language) compared to SCZ patients without alexithymia (all *p* < 0.05). Further, sum ISI score was higher in SCZ patients with alexithymia than those without alexithymia (*p* < 0.05).

**Table 1:** Socio-demographics and clinical characteristics in schizophrenic patients with and without alexithymia.

	SCZ without alexithymia	SCZ with alexithymia	X^2^/T	*p*
	*n* = 657	*n* = 320		
Age (years)	45.77 ± 12.852	46.01 ± 12.339	−192.560	0.782
Gender (M/F)	406/251	228/92	2.896	0.004^**^
Education (years)	9.69 ± 3.265	8.95 ± 2.945	10.117	0.016^*^
PANSS total score	75.64 ± 16.199	80.05 ± 16.678	−25.874	<0.001^***^
P-subscore	16.36 ± 5.526	16.41 ± 5.191	−277.049	0.885
N-subscore	20.50 ± 6.211	23.17 ± 6.790	−11.916	<0.001^***^
G-subscore	38.76 ± 8.139	40.48 ± 8.297	−17.184	0.002^*^
RBANS total score	67.51 ± 12.814	64.10 ± 11.904	20.921	<0.001^***^
Immediate memory	58.88 ± 15.124	57.24 ± 31.185	40.153	0.271
Attention	79.97 ± 15.076	77.93 ± 13.971	17.850	0.042^*^
Visuospatial	79.86 ± 16.718	77.02 ± 15.985	14.224	0.012^*^
Delayed memory	67.45 ± 18.166	63.96 ± 17.521	13.722	0.004^**^
Language	82.24 ± 13.100	78.84 ± 12.038	15.827	<0.001^***^
Sum ISI	2.50 ± 3.151	3.39 ± 4.458	−10.119	<0.001^***^

PANSS, Positive and Negative Syndrome Scale (P, positive symptom; N, negative symptom; G, general psychopathology); RBANS, Repeatable Battery for the Assessment of Neuropsychological Status. ^*^p < 0.05, ^**^p < 0.01, ^***^p < 0.001.

Table 2 showed the results of the logistic regression. It can be seen that the G-subscore of the PANSS (B = 0.074, *p* < 0.001, OR = 1.076, 95%CI, 1.035–1.120) and the TAS group (B = 0.632, *p* < 0.01, OR = 1.881, 95%CI, 1.225–2.887) were the risk factors for insomnia in SCZ patients. The gender, education, PANSS subscale (P-subscore and N-subscore) and RBANS subscale scores were not the risk factors for insomnia in SCZ patients.

**Table 2:** The risk factors for insomnia on schizophrenic patients.

	B	S.E.	*p*	OR	95%CI	
					Lower	Upper
Gender (M/F)	0.280	0.211	0.184	1.323	0.875	2.001
Education (years)	−0.020	0.032	0.532	0.980	0.920	1.044
P-subscore	−0.005	0.023	0.823	0.995	0.950	1.042
N-subscore	−0.040	0.022	0.072	0.961	0.919	1.004
G-subscore	0.074	0.020	0.000^***^	1.076	1.035	1.120
Immediate memory	0.002	0.005	0.734	1.002	0.993	1.011
Attention	−0.004	0.008	0.605	0.996	0.979	1.012
Visuospatial	0.013	0.008	0.084	1.014	0.998	1.029
Delayed memory	0.000	0.007	0.949	1.000	0.986	1.013
Language	−0.009	0.009	0.327	0.991	0.974	1.009
TAS group	0.632	0.219	0.004^**^	1.881	1.225	2.887

PANSS, Positive and Negative Syndrome Scale (P, positive symptom; N, negative symptom; G, general psychopathology); TAS group: binary classification of TAS based on recommended cut-off point (clinically significant alexithymia: TAS-20 ≥ 61; no significant alexithymia: TAS-20 < 61). ^**^p < 0.01, ^***^p < 0.001.

**Figure 2:**
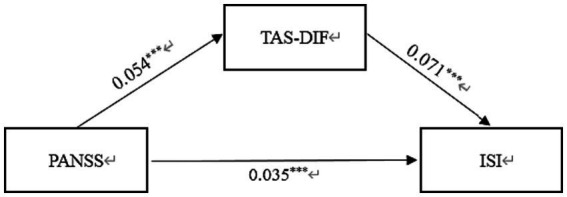
Mediation effect of TAS-DIF on the link between schizophrenia and insomnia in Chinese Patients. PANSS, Positive and Negative Syndrome scale; TAS-DIF, Toronto Alexithymia Scale-Difficulties Identifying Feelings; ISI, Insomnia Severity Index. ***p < 0.001.

In Table 3, the standardized path coefficients from schizophrenia to TAS-DIF (*β* = 0.054, *p* < 0.001) and from TAS-DIF to insomnia (*β* = 0.071, *p* < 0.001) were statistically significant. Moreover, the direct effect of Schizophrenia on Insomnia was significant (*β* = 0.035, *p* < 0.001). The total effect in this model was 0.039, and the mediating effect was 0.004, which accounted for 10.3% of the total effect. In this work, the 95% CI of the indirect effects was obtained with 5,000 bootstrap resamples (58). Figure 2 displayed the results of the mediation model.

**Table 3:** Structural model assessment of TAS-DIF on relationship between PANSS and insomnia.

	Path	Estimate	S.E.	Est. / S.E.	Standardized 95% CI		*p*-value
					Low	High	
Direct effect	PANSS→ Insomnia	0.035	0.007	4.96	0.021	0.049	<0.001	
Indirect effects	PANSS→TAS-DIF → Insomnia	0.004	0.001	4.00	0.001	0.007	<0.001	
Total effect		0.039	0.007	5.57	0.025	0.053	<0.001	
Indirect effect (%, total indirect effect/total effect)		0.004 (10.3%)						

PANSS, Positive and Negative Syndrome scale; TAS-DIF, Toronto Alexithymia Scale-Difficulties Identifying Feelings.

**Figure 3:**
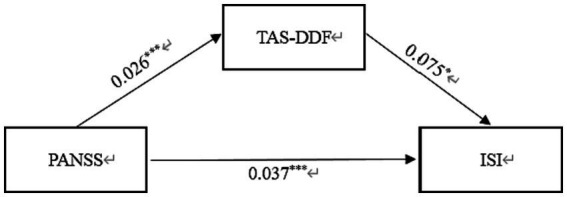
Mediation effect of TAS-DDF on the link between schizophrenia and insomnia in Chinese Patients. PANSS, Positive and Negative Syndrome scale; TAS-DDF, Toronto Alexithymia Scale-Difficulties Describing Feelings; ISI, Insomnia Severity Index. *p < 0.05, ***p < 0.001.

Similarly, Table 4 displayed the standardized path coefficients from schizophrenia to TAS-DDF (*β* = 0.026, *p* < 0.001) and these were statistically significant (*β* = 0.075, *p* < 0.05). Moreover, the direct effect of Schizophrenia on Insomnia was significant (*β* = 0.037, *p* < 0.001). The total effect in this model was 0.039, and the mediating effect was 0.002, which accounted for 5.13% of the total effect. Figure 3 exhibited the results of the mediation model.

**Table 4:** Structural model assessment of TAS-DDF on relationship between PANSS and insomnia.

	Path	Estimate	S.E.	Est. / S.E.	Standardized 95% CI		*p*-value
					Low	High	
Direct effect	PANSS→ Insomnia	0.037	0.007	5.29	0.023	0.051	<0.001
Indirect effects	PANSS→TAS-DDF → Insomnia	0.002	0.001	2.00	0.000	0.004	0.017
Total effect		0.039	0.007	5.57	0.025	0.053	<0.001
Indirect effect (%, total indirect effect/total effect)		0.002 (5.13%)					

PANSS, Positive and Negative Syndrome scale; TAS-DDF, Toronto Alexithymia Scale-Difficulties Describing Feelings.

**Figure 4:**
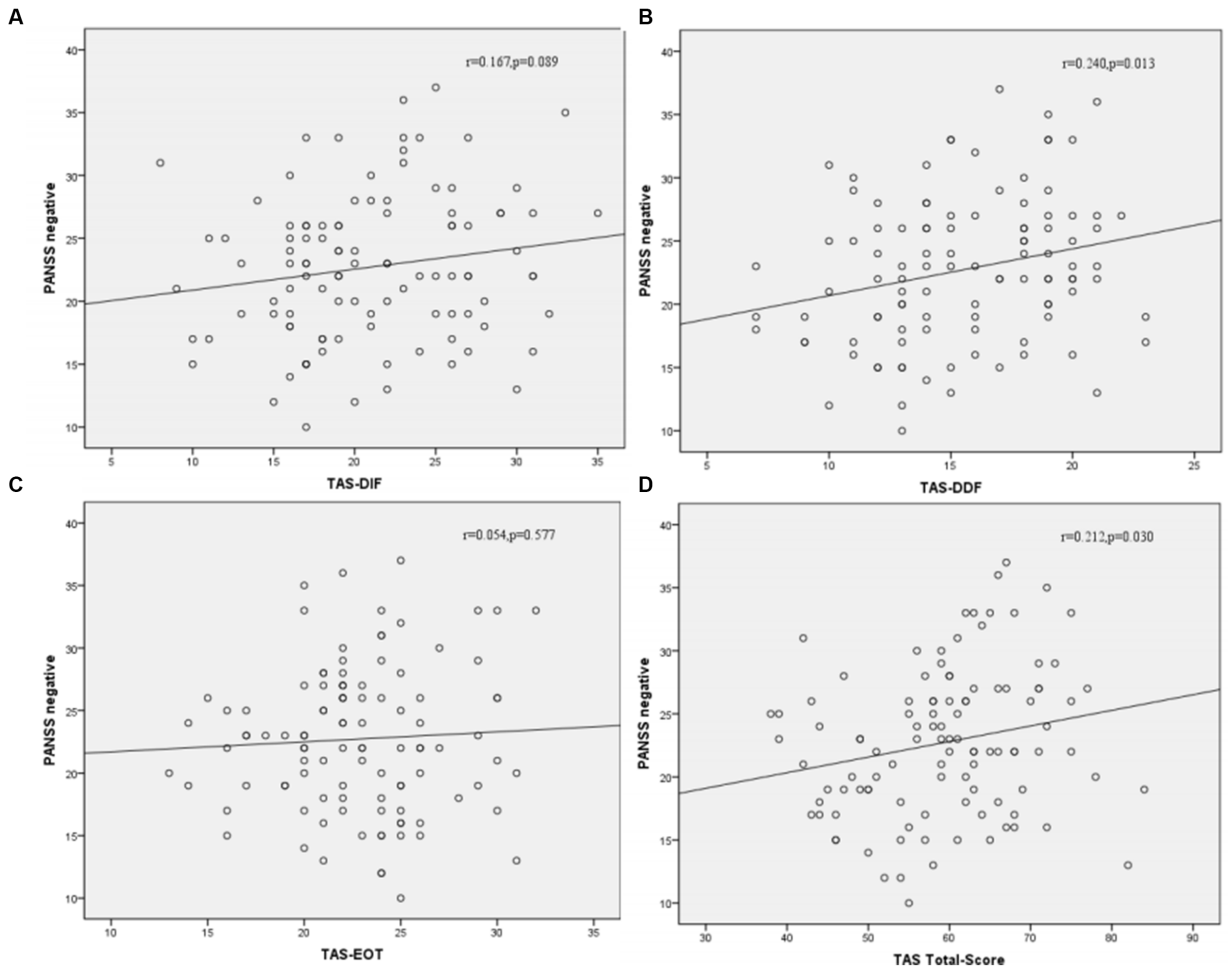
Correlation between subtypes of alexithymia [TAS-DIF **(A)**, TAS-DDF **(B)**, TAS-EOT **(C)** and TAS Total **(D)**] and PANSS negative symptoms in patients with schizophrenia. TAS-DIF, Toronto Alexithymia Scale-Difficulties Identifying Feelings, *r* = 0.167, *p* = 0.087; TAS-DDF, Toronto Alexithymia Scale-Difficulties Describing Feelings, *r* = 0.240, *p* < 0.05; TAS-EOT, Toronto Alexithymia Scale-External Oriented Thinking, *r* = 0.054, *p* = 0.577; TAS-TOTAL, Toronto Alexithymia Scale-total score, *r* = 0.212, *p* < 0.05.

**Figure 5:**
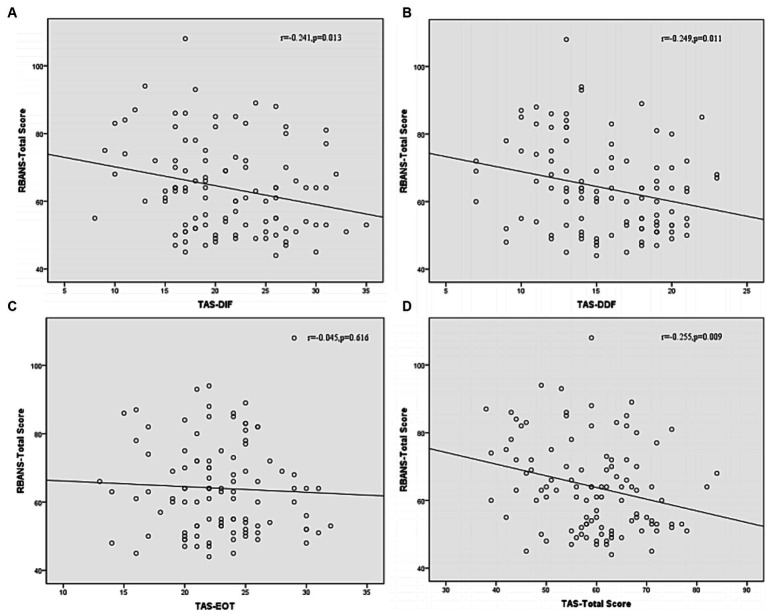
Correlation between subtypes of alexithymia [TAS-DIF **(A)**, TAS-DDF **(B)**, TAS-EOT **(C)** and TAS Total **(D)**] and RBANS total score in patients with schizophrenia. RBANS, Repeatable Battery for the Assessment of Neuropsychological Status; TAS-DIF, Toronto Alexithymia Scale-Difficulties Identifying Feelings, *r* = −0.241, *p* < 0.05; TAS-DDF, Toronto Alexithymia Scale-Difficulties Describing Feelings, *r* = −0.249, *p* < 0.05; TAS-EOT, Toronto Alexithymia Scale-External Oriented Thinking, *r* = −0.045, *p* = 0.616; TAS-TOTAL, Toronto Alexithymia Scale-total score, *r* = −0.255, *p* < 0.01.

Correlation analysis was then performed to further examine the associations between subtypes scores of alexithymia and the clinical characteristics of SCZ patients with insomnia. As shown in Figure 4, the TAS-DDF was associated with the PANSS N-subscore (*r* = 0.240, *p* < 0.05) and Figure 5 showed that both TAS-DIF (*r* = −0.241, *p* < 0.05) and TAS-DDF (*r* = −0.249, *p* < 0.05) were associated with the RBANS total score.

**Figure 6:**
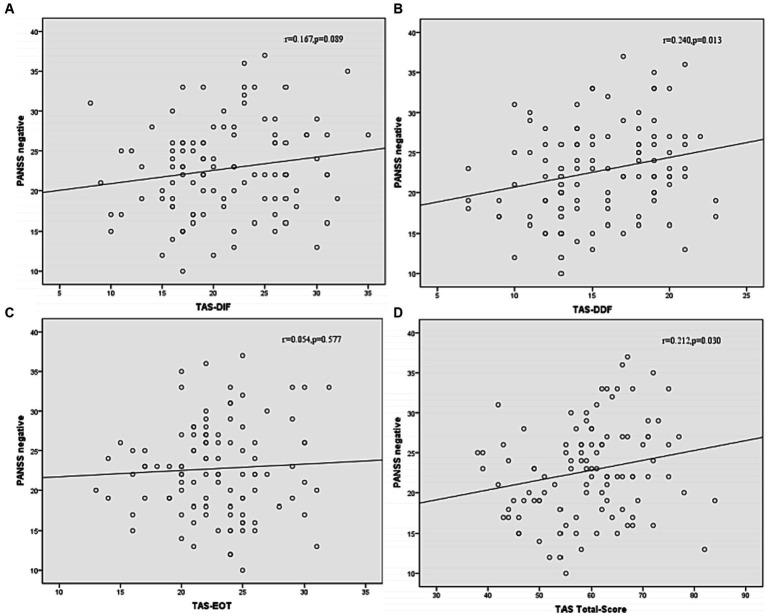
The association among ISI-Total scores and subtypes of alexithymia [TAS-DIF **(A)**, TAS-DDF **(B)**, TAS-EOT **(C)** and TAS Total **(D)**] in schizophrenia patients with insomnia. ISI, Insomnia Severity Index; TAS-DIF, Toronto Alexithymia Scale-Difficulties Identifying Feelings, *r* = 0.231, *p* < 0.05; TAS-DDF, Toronto Alexithymia Scale-Difficulties Describing Feelings, *r* = 0.205, *p* < 0.05; TAS-EOT, Toronto Alexithymia Scale-External Oriented Thinking, *r* = 0.102, *p* = 0.298; TAS-TOTAL, Toronto Alexithymia Scale-total score, *r* = 0.254, *p* < 0.01. *Bonferroni corrected *p* < 0.05/4 = 0.0125.

Figure 6 illustrates that the different dimensions of the TAS-20 scale has different associations with ISI-Total Scores in schizophrenia patients with insomnia. After Bonferroni correction (*p* < 0.05/4 = 0.0125), TAS total score was associated with the ISI-Total Scores (*r* = 0.254, *p* = 0.009).

Supplementary Table S1 shows the correlational matrix among variables of PANSS, RBANS, and TAS total score. It shows that the PANSS total score is negatively correlated with the RBANS total score, and positively correlated with the TAS total score. And, there is a negative correlation between RBANS total score and TAS total score (all *p* < 0.01).

We further subdivided three age groups for statistical analysis. Supplementary Table S2 showed that SCZ patients in young people (age < 44 years) with insomnia exhibited higher scores on the PANSS scale (total score (T-score), N-subscore and G-subscore) and higher scores on TAS scale (Total Score and TAS-DIF) than those without insomnia (all *p* < 0.05). In Supplementary Table S3, SCZ patients in middle age (age 45–59 years) with insomnia had higher scores on the PANSS scale (total score (T-score), P-subscore and G-subscore), as well as higher scores on TAS scale (Total Score, TAS-DIF and TAS-DDF) than those without insomnia (all *p* < 0.05). However, there were no statistical difference in the elderly group (age 60 years or more) (Supplementary Table S4).

## 4 Discussion

While sleep disturbances in patients with SCZ have long been a concern, very few published studies to date have explored the association between alexithymia and disturbed sleep in SCZ. This study addressed this research gap and found that insomnia was associated with psychiatric symptoms, cognitive function and alexithymic traits in Han Chinese patients diagnosed with SCZ.

This survey identified that alexithymia afflicts 32.8% of stable patients with schizophrenia, which was nearly consistent with previous studies (39, 59, 60). In contrast to past studies, prevalence rates of alexithymia have been reported at 7.1% (61), 10.0% (62), and 13% (63) in general population, which were lower than those in the SCZ group. Besides, we found that alexithymia was strongly associated with male gender and education level. The prevalence of alexithymia in the SCZ was higher in men than in women, which is in line with previous study (26). Although the education levels were associated with the alexithymia, which also was consistent with previous studies (26, 64), both gender and education level were not the risk factor for insomnia on schizophrenic patients.

In addition, the results showed a positive correlation between negative symptoms and alexithymia and a negative correlation between cognitive symptoms (attention, visuospatial, delayed memory and language) and alexithymia among the patients with schizophrenia. The underlying mechanism behind the relationship between negative symptoms and alexithymia may be that those who are unable to recognize their feelings and therefore unable to cope or regulate these feelings will be more likely to withdraw emotionally and socially (27). As for the relationship between recognition and alexithymia, our result indicated that the global ability to identify and describe emotions should be considered as a complex cognitive process requiring the integrity of several cognitive functions (i.e., attention, visuospatial, language, and executive functions) (65, 66). The appearance of these data in psychiatric populations may be the result of the gradual degradation of brain structures that regulate emotional processing and cognitive abilities (24).

The analysis to three groups of patients with different age (Supplementary Tables S2–S4) found that the elderly group does not meet the above results, and the reasons for consideration may be as follows: (1) The sample size of the elderly population is smaller than that of other groups, and we will increase the sample size in future. (2) The insomnia problem of the elderly population may be more caused by other factors, such as physical condition than emotional disorders. However, despite this, our results show consistency in the majority of the population (young and middle-aged).

Another finding of this study worthy of discussion is that alexithymia was associated with sleep disturbances. And, after Bonferroni correction, the TAS total score still is associated with insomnia. Sleep disorders may contribute to a sense of non-restoration or a feeling of mental and physical fatigue during the day (67, 68) so that patients with schizophrenia may be too tired to think about scale questions, leading to the differential results. Another explanation is that the disorder of sleep–wake system leads to dysregulation of autonomic functions (69), which may cause diminution of affect-relevant information and thus have an impact on patients’ ability to recognize their own feelings (70). Mediation analysis further demonstrates that alexithymia (both TAS-DIF and TAS-DDF) plays a partial mediating role between schizophrenic traits and sleep problems among the SCZ. This means that schizophrenic symptoms might predict directly the sleep quality of the SCZ or exert an indirect influence on sleep problems through the mediating effect of alexithymia. This result appears reasonable in line with some previous studies. For instance, negative symptoms are linked to general cognitive impairments (71), psychomotor slowing (72), and poor attention (73). These above symptoms not only may relate to alexithymia (74), but also may lead to rest-activity-rhythm (75) or rapid eye movement sleep abnormality (76). In a word, our study showed that alexithymia mediated the relationship between schizotypal traits and insomnia. Patients with symptoms of schizophrenia are more likely to have alexithymia, which may lead to increased insomnia.

There are several potential mechanisms that may explain the relationship between sleep disorders and alexithymia. One study demonstrated that brain areas involved in emotion processing (such as the amygdala, insula, anterior cingulate gyrus, etc.) exhibit abnormal neural activity in those with alexithymia (77), which might be the reason for the associated sleep disorder. And another study suggested that sleep quality might be related to emotional resonance by means of increased neural activation of a specific area within the insular cortex (78). Alternatively, high levels of alexithymia might increase difficulties in identifying and regulating emotions due to passive responses of the limbic neural circuits (including the amygdala and insular cortex) to emotional stimuli and elevated responses in the anterior cingulate cortex (ACC) (79), which may eventually result in poor sleep. Another potential mechanism that may explain how alexithymia is linked with sleep disorders involves the somatosensory system. Specifically, it has been reported that alexithymic patients have somatosensory amplification, which increases the risk of developing somatic symptoms (80). Insomnia, as a type of somatic symptom, is the internalization of psychophysiological disturbances (31). Put another way, internalized psychic conflicts and difficulty in expression brought about by increased nocturnal arousal and subsequent insomnia in individuals with alexithymia may represent a form of somatosensory amplification (33). On the other hand, insufficient sleep can increase the affective recognition and expression failure rate, as the sleep-deprived brain might suffer a connective error between subcortical overreaction and impaired higher-order prefrontal functioning (81). The worse the alexithymia, the poorer the sleep quality; thus, a vicious circle is established.

The results indicated that TAS EOT was not associated with insomnia, which is in agreement with some research results (70, 82), but not in agreement with other clinical studies (67, 83). The possible reason for this is that our experiment did not provide a detailed classification of sleep disorders, but only focused on insomnia in a broad sense. For example, De Gennaro et al. (82) revealed that EOT was not significantly correlated with sleep complaints, while they (83) reported that in three subscales of TAS-20, only EOT had a negative association with REM latency in 2002. It seems to indicate that the externally oriented thinking is not a common feature of every type of sleep disorder.

In the current study, alexithymia was found to be associated with negative symptoms. However, the N-subscore of the PANSS was not the risk factor for insomnia in SCZ patients. It is unclear whether there is a correlation among alexithymia, insomnia, and negative symptoms. We hypothesis that alexithymia may play a potentially important role in the relationship between sleep complaints and psychotic disorders by influencing negative symptoms. Specifically, both difficulties in identifying feelings and describing feelings reflect the deficits in emotional stimulation and power of imagination, aggravating negative symptoms (84), and the latter induce poor sleep (75). This hypothesis requires verification in future studies.

In summary, the findings of this study indicate that alexithymia is related to insomnia in SCZ patients. However, the mechanism underlying the association between alexithymia and SCZ-related clinical outcomes, through sleep problems, remains unknown and should be a focus of studies in the future.

Several limitations of this study should be noted. First, this was a cross-sectional study, and as such, could not examine the dynamic relationships between alexithymia, schizotypal traits and sleep. Future cohort studies should address the specific causal relationship between alexithymia and insomnia in SCZ patients. Secondly, the sample in this study was limited to people with SCZ; there was no control group. Kronholm et al. reported that alexithymic features were related to insomnia in a representative sample of Finnish adults (31). Nonetheless, the aim of the current study was to identify the factors influencing insomnia in patients with SCZ. Thirdly, this study focused on the syndrome of insomnia without considering other sleep disturbances. Future studies may wish to combine sleep encephalogram techniques with clinical symptoms in order to more deeply explore the relationship between social perceptions and sleep disturbances in SCZ, and the underlying mechanism. Besides, the correlation coefficient in mediation analysis is relatively small, which may be due to antipsychotic drug treatment. The implications of antipsychotic drugs on sleep disturbances might affect the findings. As recruited patients had already passed the onset phase and were in maintenance period treatment, they did not have long-term use of benzodiazepines, which can help them sleep well. But they had been using second-generation antipsychotic drugs for a long time, and the specific type had not been recorded. Considering many people with schizophrenia are able to sleep well with the help of antipsychotics with sedative effects, it is advisable that giving further research to exclude the interference of some certain drugs in sample on the research results. Nevertheless, in our study we find out that sleep disorders and alexithymia besides residual psychiatric symptoms are still found among a proportion of these patients in spite of medical treatment, and those need more non-drug therapy such as psychotherapy than others. As for the impact of negative symptoms on sleep, it is expected to increase research in this area in the future. Finally, psychological counseling and intervention therapy may be protective factors for preventing and alleviating symptoms. Unfortunately, this article lacks scale evaluation and data on this issue. We hope to have the opportunity to supplement or improve this aspect in the future.

## 5 Conclusion

In conclusion, SCZ patients exhibited elevated levels of alexithymia and sleep disturbances, and these symptoms were correlated. SCZ patients with dysregulated emotions were more likely to suffer from sleep disturbances, which is consistent with previous studies. The finding of a relationship between alexithymia and sleep disturbances in patients with SCZ might provide a new perspective for the treatment of SCZ patients with sleep disorders. As mentioned above, SCZ patients with insomnia and other clinical features (such as severe symptoms, emotional distress, etc.) are more likely to have a perceived poor quality of life (85), and we have reasons to believe that alexithymia is one of the poor clinical features. Thus, improving their ability to express emotions might improve their sleep quality and their quality of life in SCZ.

## Data availability statement

The original contributions presented in the study are included in the article/Supplementary material, further inquiries can be directed to the corresponding authors.

## Ethics statement

The studies involving humans were approved by the Ethics Committee of the Affiliated Kangning Hospital of Wenzhou Medical University. The studies were conducted in accordance with the local legislation and institutional requirements. The participants provided their written informed consent to participate in this study.

## Author contributions

XW, KZ, and WW conceptualized and designed the study. JL, YX, and NL recruited the participants and completed the screening assessments. JD, MW, SS, and XYW analyzed the data and performed the statistical analysis. FC, HJ, SZ, and ST wrote the first draft of the manuscript. All authors contributed to the article and approved the submitted version.

## References

[1] Marder SR Cannon TD. Schizophrenia. N Engl J Med. (2019) 381:1753–61. doi: 10.1056/NEJMra1808803 31665579

[2] McCutcheon RA Marques TR Howes OD. Schizophrenia-An Overview. JAMA Psychiatry. (2020) 77:201–10. doi: 10.1001/jamapsychiatry.2019.3360, PMID: 31664453

[3] Laskemoen JF Simonsen C Büchmann C Barrett EA Bjella T Lagerberg TV. Sleep disturbances in schizophrenia spectrum and bipolar disorders – a transdiagnostic perspective. Compr Psychiatry. (2019) 91:6–12. doi: 10.1016/j.comppsych.2019.02.006, PMID: 30856497

[4] Poe S-L Brucato G Bruno N Arndt LY Ben-David S Gill KE. Sleep disturbances in individuals at clinical high risk for psychosis. Psychiatry Res. (2017) 249:240–3. doi: 10.1016/j.psychres.2016.12.029, PMID: 28126579 PMC5893278

[5] Freeman D Taylor KM Molodynski A Waite F. Treatable clinical intervention targets for patients with schizophrenia. Schizophr Res. (2019) 211:44–50. doi: 10.1016/j.schres.2019.07.016, PMID: 31326234

[6] Wang W-L Zhou Y-Q Chai N-N Li G-H. Sleep disturbance and quality of life in clinically stable inpatients with schizophrenia in rural China. Qual Life Res. (2020) 29:2759–68. doi: 10.1007/s11136-020-02541-2, PMID: 32451984

[7] Kaskie RE Graziano B Ferrarelli F. Schizophrenia and sleep disorders: links, risks, and management challenges. Nat Sci Sleep. (2017) 9:227–39. doi: 10.2147/nss.S121076, PMID: 29033618 PMC5614792

[8] Chung K-F Poon YPY-P Ng T-K Kan C-K. Correlates of sleep irregularity in schizophrenia. Psychiatry Res. (2018) 270:705–14. doi: 10.1016/j.psychres.2018.10.064, PMID: 30551313

[9] Hofstetter JR Lysaker PH Mayeda AR. Quality of sleep in patients with schizophrenia is associated with quality of life and coping. BMC Psychiatry. (2005) 5:13. doi: 10.1186/1471-244x-5-13, PMID: 15743538 PMC554780

[10] Pocivavsek A Rowland LM. Basic neuroscience illuminates causal relationship between sleep and memory: translating to schizophrenia. Schizophr Bull. (2018) 44:7–14. doi: 10.1093/schbul/sbx151, PMID: 29136236 PMC5768044

[11] Freeman D Pugh K Vorontsova N Southgate L. Insomnia and paranoia. Schizophr Res. (2009) 108:280–4. doi: 10.1016/j.schres.2008.12.001, PMID: 19097752 PMC2697325

[12] Reeve S Nickless A Sheaves B Hodgekins J Stewart SLK Gumley A. Sleep duration and psychotic experiences in patients at risk of psychosis: a secondary analysis of the EDIE-2 trial. Schizophr Res. (2019) 204:326–33. doi: 10.1016/j.schres.2018.08.006, PMID: 30121185 PMC6406020

[13] Taylor MJ Gregory AM Freeman D Ronald A. Do sleep disturbances and psychotic-like experiences in adolescence share genetic and environmental influences? J Abnorm Psychol. (2015) 124:674–84. doi: 10.1037/abn0000057 25938536 PMC4532318

[14] Manoach DS Stickgold R. Abnormal sleep spindles, memory consolidation, and schizophrenia. Annu Rev Clin Psychol. (2019) 15:451–79. doi: 10.1146/annurev-clinpsy-050718-095754, PMID: 30786245 PMC7307009

[15] Nebes RD Buysse DJ Halligan EM Houck PR Monk TH. Self-reported sleep quality predicts poor cognitive performance in healthy older adults. J Gerontol B Psychol Sci Soc Sci. (2009) 64:180–7. doi: 10.1093/geronb/gbn037, PMID: 19204069 PMC2655169

[16] Li SX Lam SP Zhang J Mandy Wai Man Y Chan JWY Chan CSY. Sleep disturbances and suicide risk in an 8-year longitudinal study of schizophrenia-Spectrum disorders. Sleep. (2016) 39:1275–82. doi: 10.5665/sleep.5852, PMID: 27091530 PMC4863217

[17] Nemiah JC Sifneos PE. Psychosomatic illness: a problem in communication. Psychother Psychosom. (1970) 18:154–60. doi: 10.1159/000286074 5520658

[18] Chen J Ting X Jing J Chan RCK. Alexithymia and emotional regulation: a cluster analytical approach. BMC Psychiatry. (2011) 11:33. doi: 10.1186/1471-244x-11-33, PMID: 21345180 PMC3050802

[19] Iskric A Ceniti AK Bergmans Y McInerney S Rizvi SJ. Alexithymia and self-harm: a review of nonsuicidal self-injury, suicidal ideation, and suicide attempts. Psychiatry Res. (2020) 288:112920. doi: 10.1016/j.psychres.2020.112920, PMID: 32279008

[20] Taylor GJ. Alexithymia: concept, measurement, and implications for treatment. Am J Psychiatry. (1984) 141:725–32. doi: 10.1176/ajp.141.6.725 6375397

[21] Aaron RV Benson TL Park S. Investigating the role of alexithymia on the empathic deficits found in schizotypy and autism spectrum traits. Pers Individ Dif. (2015) 77:215–20. doi: 10.1016/j.paid.2014.12.032 29472731 PMC5820003

[22] Heshmati R Jafari E Hoseinifar J Ahmadi M. Comparative study of alexithymia in patients with schizophrenia spectrum disorders, non-psychotic disorders and normal people. Procedia Soc Behav Sci. (2010) 5:1084–9. doi: 10.1016/j.sbspro.2010.07.240

[23] O'Driscoll C Laing J Mason O. Cognitive emotion regulation strategies, alexithymia and dissociation in schizophrenia, a review and meta-analysis. Clin Psychol Rev. (2014) 34:482–95. doi: 10.1016/j.cpr.2014.07.002, PMID: 25105273

[24] Gawęda Ł Krężołek M. Cognitive mechanisms of alexithymia in schizophrenia: investigating the role of basic neurocognitive functioning and cognitive biases. Psychiatry Res. (2019) 271:573–80. doi: 10.1016/j.psychres.2018.12.023 30554105

[25] Fogley R Warman D Lysaker PH. Alexithymia in schizophrenia: associations with neurocognition and emotional distress. Psychiatry Res. (2014) 218:1–6. doi: 10.1016/j.psychres.2014.04.020, PMID: 24794152

[26] Yi Y Huang Y Jiang R Chen Q Yang M Li H. The percentage and clinical correlates of alexithymia in stable patients with schizophrenia. Eur Arch Psychiatry Clin Neurosci. (2023) 273:679–86. doi: 10.1007/s00406-022-01492-8, PMID: 36239818 PMC10085932

[27] van't Wout M Aleman A Bermond B Kahn RS. No words for feelings: alexithymia in schizophrenia patients and first-degree relatives. Compr Psychiatry. (2007) 48:27–33. doi: 10.1016/j.comppsych.2006.07.003, PMID: 17145278

[28] Kubota M Miyata J Hirao K Fujiwara H Kawada R Fujimoto S. Alexithymia and regional gray matter alterations in schizophrenia. Neurosci Res. (2011) 70:206–13. doi: 10.1016/j.neures.2011.01.019, PMID: 21300113

[29] Kubota M Miyata J Sasamoto A Kawada R Fujimoto S Tanaka Y. Alexithymia and reduced white matter integrity in schizophrenia: a diffusion tensor imaging study on impaired emotional self-awareness. Schizophr Res. (2012) 141:137–43. doi: 10.1016/j.schres.2012.08.026, PMID: 22986045

[30] Alimoradi Z Majd NR Broström A Tsang HWH Singh P Ohayon MM. Is alexithymia associated with sleep problems? A systematic review and meta-analysis. Neurosci Biobehav Rev. (2022) 133:104513. doi: 10.1016/j.neubiorev.2021.12.036, PMID: 34958823

[31] Kronholm E Partonen T Salminen JK Mattila AK Joukamaa M. Alexithymia, depression and sleep disturbance symptoms. Psychother Psychosom. (2008) 77:63–5. doi: 10.1159/000110063 18087211

[32] Honkalampi K Saarinen P Hintikka J Virtanen V Viinamäki H. Factors associated with alexithymia in patients suffering from depression. Psychother Psychosom. (1999) 68:270–5. doi: 10.1159/000012343 10516532

[33] Bauermann TM Parker JDA Taylor GJ. Sleep problems and sleep hygiene in young adults with alexithymia. Personal Individ Differ. (2008) 45:318–22. doi: 10.1016/j.paid.2008.04.019

[34] Parker JD Bauermann TM Smith CT. Alexithymia and impoverished dream content: evidence from rapid eye movement sleep awakenings. Psychosom Med. (2000) 62:486–91. doi: 10.1097/00006842-200007000-00006 10949093

[35] Gujar N McDonald SA Nishida M Walker MP. A role for REM sleep in recalibrating the sensitivity of the human brain to specific emotions. Cereb Cortex. (2011) 21:115–23. doi: 10.1093/cercor/bhq064 20421251 PMC3000566

[36] Scarpelli S Bartolacci C D'Atri A Gorgoni M De Gennaro L. The functional role of dreaming in emotional processes. Front Psychol. (2019) 10:459. doi: 10.3389/fpsyg.2019.00459, PMID: 30930809 PMC6428732

[37] Nielsen T. The stress acceleration hypothesis of nightmares. Front Neurol. (2017) 8:201. doi: 10.3389/fneur.2017.00201, PMID: 28620339 PMC5451501

[38] Rosen IM Gimotty PA Shea JA Bellini LM. Evolution of sleep quantity, sleep deprivation, mood disturbances, empathy, and burnout among interns. Acad Med. (2006) 81:82–5. doi: 10.1097/00001888-200601000-00020, PMID: 16377826

[39] Ma Q Zhang X Zou L. The mediating effect of alexithymia on the relationship between schizotypal traits and sleep problems among college students. Front Psych. (2020) 11:153. doi: 10.3389/fpsyt.2020.00153, PMID: 32194461 PMC7064435

[40] Rehman A Gumley A Biello S. Sleep quality and paranoia: the role of alexithymia, negative emotions and perceptual anomalies. Psychiatry Res. (2018) 259:216–22. doi: 10.1016/j.psychres.2017.09.066, PMID: 29080493

[41] Pei C Fan C Luo H Bai A Ni S Luo M. Sleep problems in adolescents with depression: role of childhood trauma, alexithymia, rumination, and self-esteem. J Affect Disord. (2023) 338:83–91. doi: 10.1016/j.jad.2023.05.095, PMID: 37269886

[42] Leucht S Tardy M Komossa K Heres S Kissling W Salanti G. Antipsychotic drugs versus placebo for relapse prevention in schizophrenia: a systematic review and meta-analysis. Lancet. (2012) 379:2063–71. doi: 10.1016/S0140-6736(12)60239-6, PMID: 22560607

[43] Shimomura Y Kikuchi Y Suzuki T Uchida H Mimura M Takeuchi H. Antipsychotic treatment in the maintenance phase of schizophrenia: an updated systematic review of the guidelines and algorithms. Schizophr Res. (2020) 215:8–16. doi: 10.1016/j.schres.2019.09.013, PMID: 31784340

[44] Zhang S Cui Y Weng Z Gong X Chen M Zhong B. Changes on the disease pattern of primary colorectal cancers in southern China: a retrospective study of 20 years. Int J Color Dis. (2009) 24:943–9. doi: 10.1007/s00384-009-0726-y, PMID: 19424708

[45] Bagby RM Parker JDA Taylor GJ. The twenty-item Toronto alexithymia scale--I. Item selection and cross-validation of the factor structure. J Psychosom Res. (1994) 38:23–32. doi: 10.1016/0022-3999(94)90005-1 8126686

[46] Bagby RM Taylor GJ Parker JDA. The twenty-item Toronto alexithymia scale--II. Convergent, discriminant, and concurrent validity. J Psychosom Res. (1994) 38:33–40. doi: 10.1016/0022-3999(94)90006-x 8126688

[47] Veirman E Van Ryckeghem DML Verleysen G De Paepe AL Crombez G. What do alexithymia items measure? A discriminant content validity study of the Toronto-alexithymia-scale-20. PeerJ. (2021) 9:e11639. doi: 10.7717/peerj.11639, PMID: 34249500 PMC8253111

[48] Kay SR Fiszbein A Opler LA. The positive and negative syndrome scale (PANSS) for schizophrenia. Schizophr Bull. (1987) 13:261–76. doi: 10.1093/schbul/13.2.261 3616518

[49] Raudeberg R Karr JE Iverson GL Hammar Å. Examining the repeatable battery for the assessment of neuropsychological status validity indices in people with schizophrenia spectrum disorders. Clin Neuropsychol. (2023) 37:101–18. doi: 10.1080/13854046.2021.1876169, PMID: 33522847

[50] Duff K Randolph C Boxer AL. Cognitive decline on the repeatable battery for the assessment of neuropsychological status in progressive supranuclear palsy. Clin Neuropsychol. (2020) 34:529–40. doi: 10.1080/13854046.2019.1670865 31559910 PMC7083686

[51] Tsatali M Fotiadou F Giaglis G Tsolaki M. The repeatable battery for the assessment of the neuropsychological status (RBANS): a diagnostic validity study in Greek elderly. Aging Clin Exp Res. (2019) 31:1305–12. doi: 10.1007/s40520-018-1076-9, PMID: 30471005

[52] Bastien CH Vallières A Morin CM. Validation of the insomnia severity index as an outcome measure for insomnia research. Sleep Med. (2001) 2:297–307. doi: 10.1016/s1389-9457(00)00065-4 11438246

[53] Miller BJ McCall WV Xia L Zhang Y Li W Yao X. Insomnia, suicidal ideation, and psychopathology in Chinese patients with chronic schizophrenia. Prog Neuro-Psychopharmacol Biol Psychiatry. (2021) 111:110202. doi: 10.1016/j.pnpbp.2020.110202, PMID: 33285266

[54] Morin CM Belleville G Bélanger L Ivers H. The insomnia severity index: psychometric indicators to detect insomnia cases and evaluate treatment response. Sleep. (2011) 34:601–8. doi: 10.1093/sleep/34.5.601, PMID: 21532953 PMC3079939

[55] Morriss RK Wearden AJ Mullis R. Exploring the validity of the Chalder fatigue scale in chronic fatigue syndrome. J Psychosom Res. (1998) 45:411–7. doi: 10.1016/s0022-3999(98)00022-1, PMID: 9835234

[56] Preacher KJ Hayes AF. SPSS and SAS procedures for estimating indirect effects in simple mediation models. Behav Res Methods Instrum Comput. (2004) 36:717–31. doi: 10.3758/bf03206553, PMID: 15641418

[57] Guillén V Santos B Muñoz P Fernández B de Corres E Fernández IP. Toronto alexithymia scale for patients with eating disorder: of performance using the non-parametric item response theory. Compr Psychiatry. (2014) 55:1285–91. doi: 10.1016/j.comppsych.2014.03.020, PMID: 24791683

[58] Hayes AF. Introduction to mediation, moderation, and conditional process analysis. J Educ Meas. (2013) 51:335–7.

[59] Cedro A Kokoszka A Popiel A Narkiewicz-Jodko W. Alexithymia in schizophrenia: an exploratory study. Psychol Rep. (2001) 89:95–8. doi: 10.2466/pr0.2001.89.1.95 11729558

[60] McGillivray L Becerra R Harms C. Prevalence and demographic correlates of alexithymia: a comparison between Australian psychiatric and community samples. J Clin Psychol. (2017) 73:76–87. doi: 10.1002/jclp.22314, PMID: 27129142

[61] Kokkonen P Karvonen JT Veijola J La Ksy K Jokelainen J Ja rvelin M-R. Prevalence and sociodemographic correlates of alexithymia in a population sample of young adults. Compr Psychiatry. (2001) 42:471–6. doi: 10.1053/comp.2001.27892 11704938

[62] Holmes A Marella P Rodriguez C Ii DG Goerlich KS. Alexithymia and cutaneous disease morbidity: a systematic review. Dermatology. (2022) 238:1120–9. doi: 10.1159/000524736, PMID: 35636409

[63] Salminen JK Saarijärvi S Äärelä E Toikka T Kauhanen J. Prevalence of alexithymia and its association with sociodemographic variables in the general population of Finland. J Psychosom Res. (1999) 46:75–82. doi: 10.1016/s0022-3999(98)00053-1 10088984

[64] Chen L Linna X You W Zhang X Ling N. Prevalence and associated factors of alexithymia among adult prisoners in China: a cross-sectional study. BMC Psychiatry. (2017) 17:287. doi: 10.1186/s12888-017-1443-7 28768497 PMC5541430

[65] Assogna F Pontieri FE Cravello L Peppe A Pierantozzi M Stefani A. Intensity-dependent facial emotion recognition and cognitive functions in Parkinson's disease. J Int Neuropsychol Soc. (2010) 16:867–76. doi: 10.1017/s1355617710000755, PMID: 20663240

[66] Cecchetto C Aiello M D'Amico D Cutuli D Cargnelutti D Eleopra R. Facial and bodily emotion recognition in multiple sclerosis: the role of alexithymia and other characteristics of the disease. J Int Neuropsychol Soc. (2014) 20:1004–14. doi: 10.1017/s1355617714000939, PMID: 25373767

[67] Bazydlo R Lumley MA Roehrs T. Alexithymia and polysomnographic measures of sleep in healthy adults. Psychosom Med. (2001) 63:56–61. doi: 10.1097/00006842-200101000-00007, PMID: 11211065

[68] Vandekerckhove M Cluydts R. The emotional brain and sleep: an intimate relationship. Sleep Med Rev. (2010) 14:219–26. doi: 10.1016/j.smrv.2010.01.002, PMID: 20363166

[69] Kim H Jung HR Kim JB Kim D-J. Autonomic dysfunction in sleep disorders: from neurobiological basis to potential therapeutic approaches. J Clin Neurol. (2022) 18:140–51. doi: 10.3988/jcn.2022.18.2.140, PMID: 35274834 PMC8926769

[70] Godin I Montplaisir J Gagnon J-F Nielsen T. Alexithymia associated with nightmare distress in idiopathic REM sleep behavior disorder. Sleep. (2013) 36:1957–62. doi: 10.5665/sleep.3238, PMID: 24293771 PMC3825446

[71] Deserno L Heinz A Schlagenhauf F. Computational approaches to schizophrenia: a perspective on negative symptoms. Schizophr Res. (2017) 186:46–54. doi: 10.1016/j.schres.2016.10.004, PMID: 27986430

[72] Heinz A Knable MB Coppola R Gorey JG Jones DW Lee K-S. Psychomotor slowing, negative symptoms and dopamine receptor availability--an IBZM SPECT study in neuroleptic-treated and drug-free schizophrenic patients. Schizophr Res. (1998) 31:19–26. doi: 10.1016/s0920-9964(98)00003-6 9633833

[73] Navalón P Sahuquillo-Leal R Moreno-Giménez A Salmerón L Benavent P Sierra P. Attentional engagement and inhibitory control according to positive and negative symptoms in schizophrenia: an emotional antisaccade task. Schizophr Res. (2022) 239:142–50. doi: 10.1016/j.schres.2021.11.044, PMID: 34891078

[74] Nkam I Langlois-Thery S Dollfus S Petit M. Negative symptoms, depression, anxiety and alexithymia in DSM III-R schizophrenic patients. Encéphale. (1997) 23:267–72. PMID: 9417392

[75] Mayeli A LaGoy AD Smagula SF Wilson JD Zarbo C Rocchetti M. Shared and distinct abnormalities in sleep-wake patterns and their relationship with the negative symptoms of schizophrenia Spectrum disorder patients. Mol Psychiatry. (2023) 28:2049–57. doi: 10.1038/s41380-023-02050-x, PMID: 37055512

[76] Tandon R Shipley JE Eiser AS Greden JF. Association between abnormal REM sleep and negative symptoms in schizophrenia. Psychiatry Res. (1989) 27:359–61. doi: 10.1016/0165-1781(89)90151-0, PMID: 2710873

[77] Moriguchi Y Komaki G. Neuroimaging studies of alexithymia: physical, affective, and social perspectives. Biopsychosoc Med. (2013) 7:8. doi: 10.1186/1751-0759-7-8, PMID: 23537323 PMC3621096

[78] Guadagni V Burles F Ferrara M Iaria G. Sleep quality and its association with the insular cortex in emotional empathy. Eur J Neurosci. (2018) 48:2288–300. doi: 10.1111/ejn.14124, PMID: 30118565

[79] van der Velde J Servaas MN Goerlich KS Bruggeman R Horton P Costafreda SG. Neural correlates of alexithymia: a meta-analysis of emotion processing studies. Neurosci Biobehav Rev. (2013) 37:1774–85. doi: 10.1016/j.neubiorev.2013.07.008, PMID: 23886515

[80] Nakao M Takeuchi T. Alexithymia and somatosensory amplification link perceived psychosocial stress and somatic symptoms in outpatients with psychosomatic illness. J Clin Med. (2018) 7:112. doi: 10.3390/jcm7050112, PMID: 29748483 PMC5977151

[81] Goldstein AN Walker MP. The role of sleep in emotional brain function. Annu Rev Clin Psychol. (2014) 10:679–708. doi: 10.1146/annurev-clinpsy-032813-153716 24499013 PMC4286245

[82] De Gennaro L Martina M Curcio G Ferrara M. The relationship between alexithymia, depression, and sleep complaints. Psychiatry Res. (2004) 128:253–8. doi: 10.1016/j.psychres.2004.05.023, PMID: 15541782

[83] De Gennaro L Ferrara M Curcio G Cristiani R Lombardo C Bertini M. Are polysomnographic measures of sleep correlated to alexithymia? A study on laboratory-adapted sleepers. J Psychosom Res. (2002) 53:1091–5. doi: 10.1016/s0022-3999(02)00342-2 12479991

[84] İnanç L Sevinç E Semiz ÜB. Relationship between alexithymia, depression and the negative symptoms in schizophrenia with and without deficit syndrome. Turk Psikiyatri Derg. (2019) 30:225–35. PMID: 32594483

[85] Ritsner M Kurs R Ponizovsky A Hadjez J. Perceived quality of life in schizophrenia: relationships to sleep quality. Qual Life Res. (2004) 13:783–91. doi: 10.1023/B:QURE.0000021687.18783.d6, PMID: 15129888

