# Supplementary material for: Alexithymia is associated with insomnia in Chinese patients with schizophrenia
Source: Front Psychiatry. 2023 Dec 15;14:1252763. doi: 10.3389/fpsyt.2023.1252763 (PMC10757626; doi:10.3389/fpsyt.2023.1252763)
Supplement: Supplementary file 1 [file Data_Sheet_1.docx]

Supplementary table 1 Pearson’s correlation matrix among variables of interest.

|  | PANSS total score | RBANS total score | TAS total score |
| --- | --- | --- | --- |
| PANSS total score | 1 |  |  |
| RBANS total score | -.378^**^ | 1 |  |
| TAS total score | .176^**^ | -.212^**^ | 1 |

Note: PANSS, Positive and Negative Syndrome Scale; RBANS, Repeatable Battery for the Assessment of Neuropsychological Status; TAS, Toronto Alexithymia Scale. **P< 0.01.

Supplementary table 2 Socio-demographics and clinical characteristics in schizophrenic patients with and without insomnia in youth group under 44 years old.

|  | **SCZ**  **without insomnia** | **SCZ**  **with insomnia** | **T** | **P** |
| --- | --- | --- | --- | --- |
|  | n=382 | n=49 |  |  |
| Age(years) | 33.9±6.8 | 33±6.2 | 0.89 | 0.376 |
| PANSS total score | 75.7±17.6 | 83.4±12.5 | -2.94 | 0.004^**^ |
| P-subscore | 16.5±5.8 | 17.9±5.5 | -1.56 | 0.120 |
| N-subscore | 20.6±6.8 | 22.7±5.6 | -2.11 | 0.035^*^ |
| G-subscore | 38.6±8.8 | 42.7±6.5 | -3.21 | 0.001^**^ |
| RBANS total score | 68.0±13.5 | 65.0±14.5 | 1.48 | 0.139 |
| Immediate memory | 61.5±30.2 | 57.7±15.2 | 0.87 | 0.39 |
| Attention | 81.5±15.5 | 77.1±17.0 | 1.86 | 0.063 |
| Visuospatial | 79.0±16.9 | 77.9±19.3 | 0.43 | 0.668 |
| Delayed memory | 67.8±19.1 | 64.6±21.5 | 1.09 | 0.275 |
| Language | 81.8±14.3 | 78.3±14.0 | 1.62 | 0.106 |
| TAS total score | 55.5±10.2 | 59.8±10.3 | -2.73 | 0.007^**^ |
| F1 | 18.8±6.0 | 21.7±5.8 | -3.12 | 0.002^**^ |
| F2 | 14.5±3.6 | 15.4±3.8 | -1.66 | 0.098 |
| F3 | 22.2±4.2 | 22.7±4.1 | -0.796 | 0.426 |

Note: PANSS, Positive and Negative Syndrome Scale (P, positive symptom; N, negative symptom; G, general psychopathology); RBANS, Repeatable Battery for the Assessment of Neuropsychological Status; TAS, Toronto Alexithymia Scale (F1, difficulty identifying feelings; F2, difficulty describing feelings; F3, externally oriented thinking). ^*^P<0.05, ^**^P<0.01.

Supplementary table 3 Socio-demographics and clinical characteristics in schizophrenic patients with and without insomnia in middle aged group aged 45 to 60.

|  | **SCZ**  **without insomnia** | **SCZ**  **with insomnia** | **T** | **P** |
| --- | --- | --- | --- | --- |
|  | n=348 | n=39 |  |  |
| Age(years) | 51.8±4.1 | 51.8±3.8 | -0.07 | 0.944 |
| PANSS total score | 75.7±15.8 | 85.1±16.9 | -3.48 | 0.001^**^ |
| P-subscore | 16.1±5.2 | 18.1±5.3 | -2.25 | 0.025^*^ |
| N-subscore | 21.2±6.4 | 23.1±5.9 | -1.70 | 0.091 |
| G-subscore | 38.4±7.6 | 43.9±9.3 | -4.20 | <0.001^***^ |
| RBANS total score | 66.4±12.2 | 64.4±13.7 | 0.97 | 0.335 |
| Immediate memory | 57.1±14.3 | 57.7±14.2 | -0.27 | 0.786 |
| Attention | 78.9±14.4 | 78.2±14.2 | 0.29 | 0.770 |
| Visuospatial | 80.2±16.4 | 78.2±17.4 | 0.74 | 0.463 |
| Delayed memory | 66.4±17.2 | 62.3±16.7 | 1.40 | 0.162 |
| Language | 81.4±11.4 | 77.8±15.1 | 1.82 | 0.069 |
| TAS total score | 55.7±9.3 | 59.7±10.0 | -2.53 | 0.012^*^ |
| F1 | 18.8±5.6 | 21.2±5.7 | -2.50 | 0.013^*^ |
| F2 | 14.3±3.7 | 15.8±4.2 | -2.29 | 0.022^*^ |
| F3 | 22.5±4.3 | 22.7±4.2 | -0.27 | 0.789 |

Note: PANSS, Positive and Negative Syndrome Scale (P, positive symptom; N, negative symptom; G, general psychopathology); RBANS, Repeatable Battery for the Assessment of Neuropsychological Status; TAS, Toronto Alexithymia Scale (F1, difficulty identifying feelings; F2, difficulty describing feelings; F3, externally oriented thinking). ^*^P<0.05, ^**^P<0.01, ^***^P<0.001.

Supplementary table 4 Socio-demographics and clinical characteristics in schizophrenic patients with and without insomnia in elderly group over 60 years old.

|  | **SCZ**  **without insomnia** | **SCZ**  **with insomnia** | **T** | **P** |
| --- | --- | --- | --- | --- |
|  | n=142 | n=17 |  |  |
| Age(years) | 64.1±3.1 | 64.8±3.5 | -0.83 | 0.406 |
| PANSS total score | 79.4±15 | 81±13.5 | -0.44 | 0.664 |
| P-subscore | 15.6±4.8 | 16.8±5.2 | -1.00 | 0.318 |
| N-subscore | 22.9±6.2 | 22±6.4 | 0.53 | 0.597 |
| G-subscore | 40.9±7.5 | 42.2±7.2 | -0.70 | 0.488 |
| RBANS total score | 63.9±9.6 | 60.8±9.1 | 1.29 | 0.200 |
| Immediate memory | 54.3±10.4 | 50.6±9.4 | 1.39 | 0.168 |
| Attention | 76.3±12.0 | 71.2±13.7 | 1.64 | 0.103 |
| Visuospatial | 76.2±14.4 | 79.1±15.1 | -0.80 | 0.424 |
| Delayed memory | 64.6±16 | 60.2±15 | 1.09 | 0.279 |
| Language | 80.7±10.5 | 78.9±15.5 | 0.62 | 0.534 |
| TAS total score | 57.4±8.7 | 57.3±9.0 | 0.06 | 0.952 |
| F1 | 18.7±6.0 | 18.7±5.8 | 0.02 | 0.986 |
| F2 | 15.0±3.7 | 15.1±3.0 | -0.10 | 0.921 |
| F3 | 23.7±4.1 | 23.5±3.3 | 0.20 | 0.845 |

Note: PANSS, Positive and Negative Syndrome Scale (P, positive symptom; N, negative symptom; G, general psychopathology); RBANS, Repeatable Battery for the Assessment of Neuropsychological Status; TAS, Toronto Alexithymia Scale (F1, difficulty identifying feelings; F2, difficulty describing feelings; F3, externally oriented thinking).

Supplementary table 5 Structural model assessment

|  | Path | Estimate | S.E. | Est. / S.E. | Standardized 95% CI  Low High | | P-value |
| --- | --- | --- | --- | --- | --- | --- | --- |
| Direct Effect | Insomnia→ TAS-DIF | 0.199 | 0.051 | 3.90 | 0.100 | 0.298 | <0.001 |
| Indirect Effects | Insomnia→RBANS→TAS-TIF | 0.020 | 0.009 | 2.22 | 0.006 | 0.040 | <0.001 |
| Total Effect | | 0.219 | 0.051 | 4.29 | 0.120 | 0.319 | <0.001 |
| Indirect effect  (%, total indirect effect/total effect) | | 0.020  (9.13%) |  |  |  |  |  |

**Note:** RBANS, Repeatable Battery for the Assessment of Neuropsychological Status; TAS-DIF, Toronto Alexithymia Scale-Difficulties Identifying Feelings.

Supplementary table 6 Structural model assessment

|  | Path | Estimate | S.E. | Est. / S.E. | Standardized 95% CI  Low High | | P-value |
| --- | --- | --- | --- | --- | --- | --- | --- |
| Direct Effect | Insomnia→ TAS-DDF | 0.081 | 0.032 | 2.53 | 0.018 | 0.144 | 0.012 |
| Indirect Effects | Insomnia→RBANS→TAS-DDF | 0.015 | 0.006 | 2.50 | 0.005 | 0.028 | <0.001 |
| Total Effect | | 0.096 | 0.032 | 3.00 | 0.033 | 0.160 | 0.003 |
| Indirect effect  (%, total indirect effect/total effect) | | 0.015  (15.63%) |  |  |  |  |  |

**Note:** RBANS, Repeatable Battery for the Assessment of Neuropsychological Status; TAS-DDF, Toronto Alexithymia Scale-Difficulties Describing Feelings.

Supplementary Figure 1 Normal distribution test for each variable: QQ plot


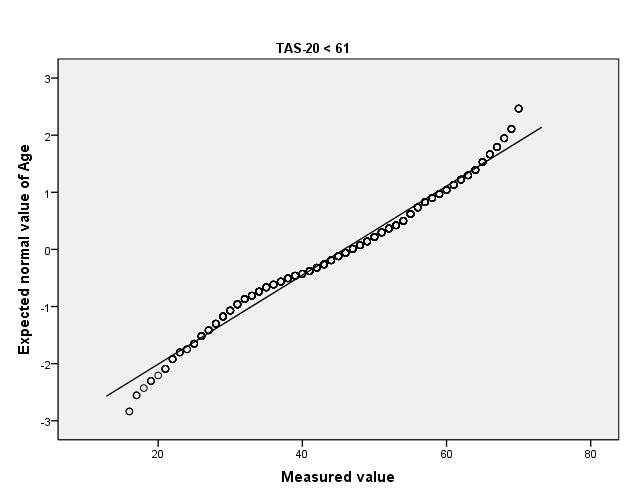

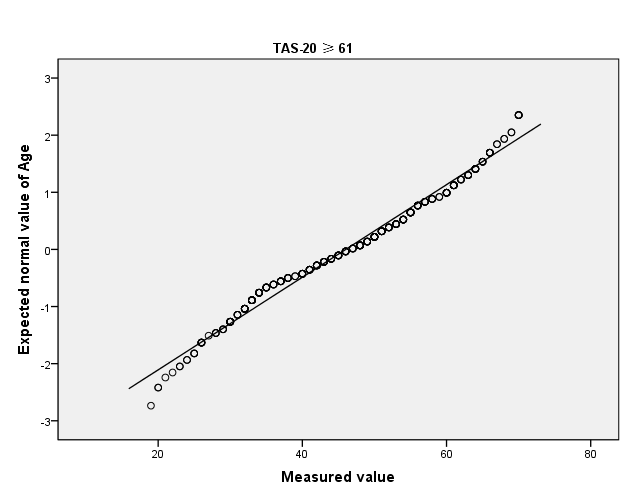


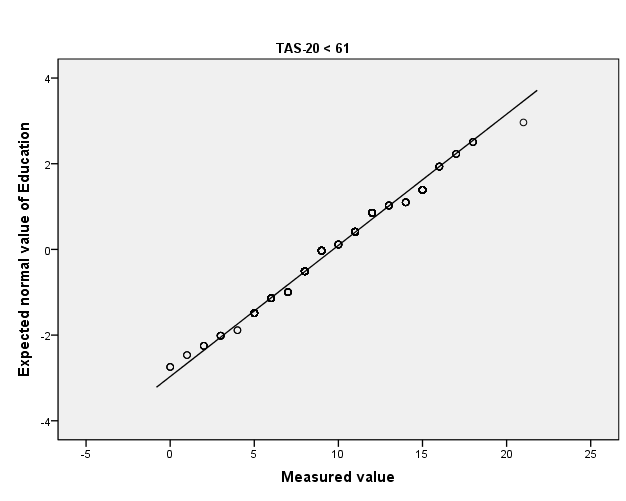

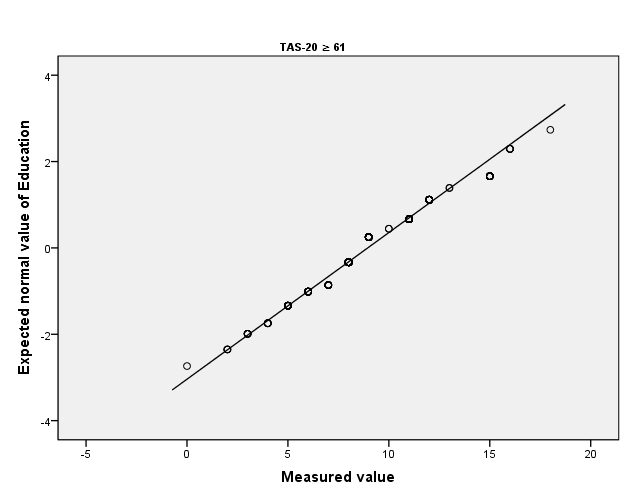


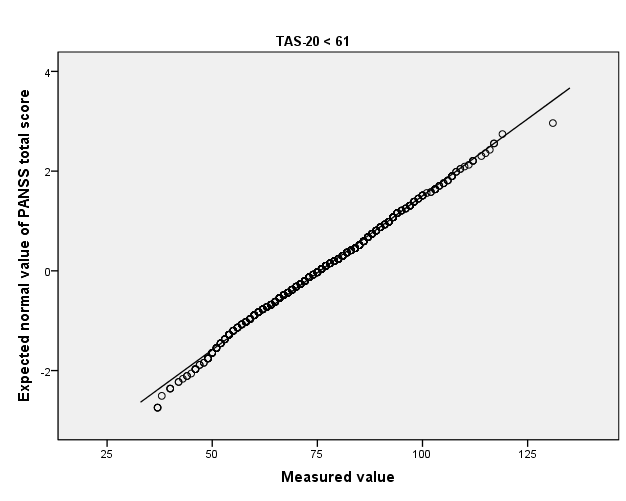

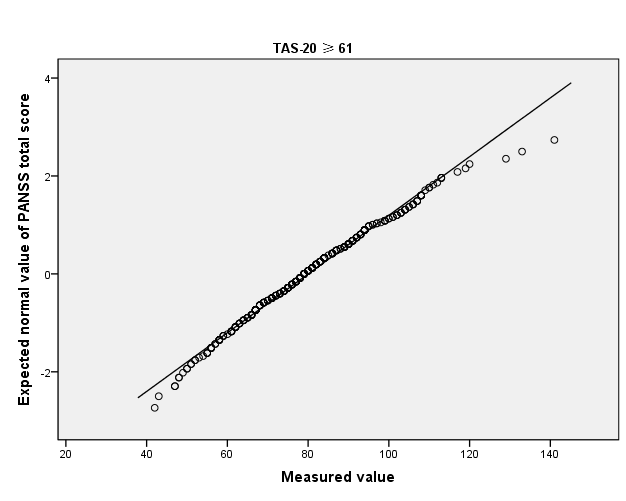


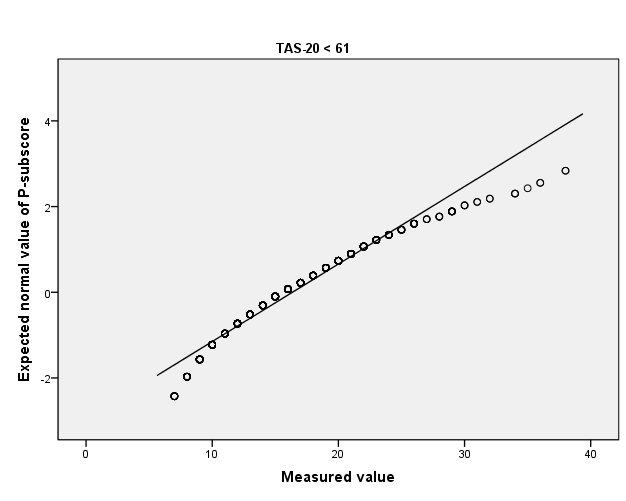

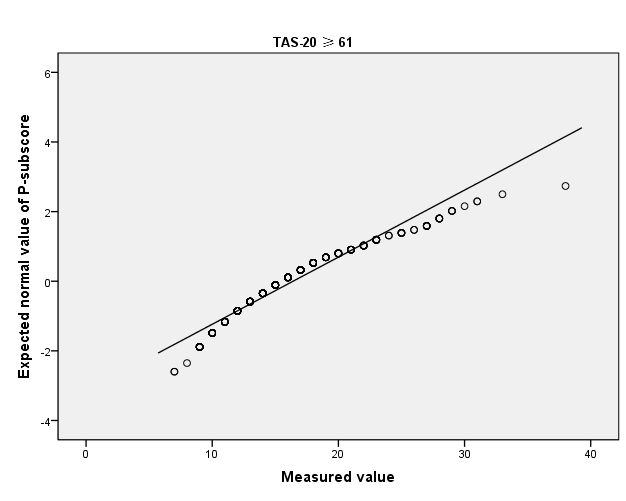


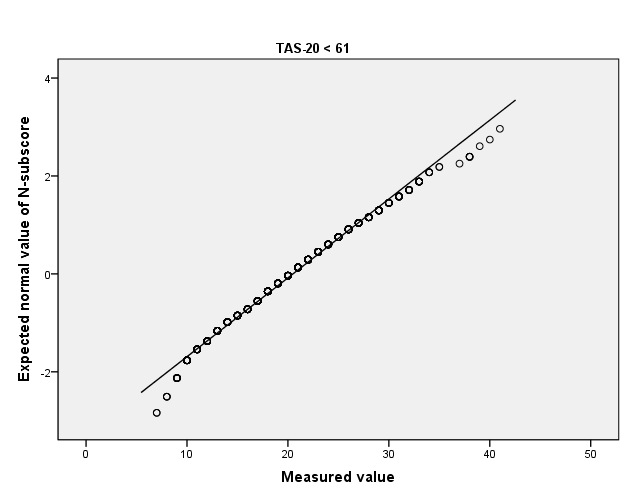

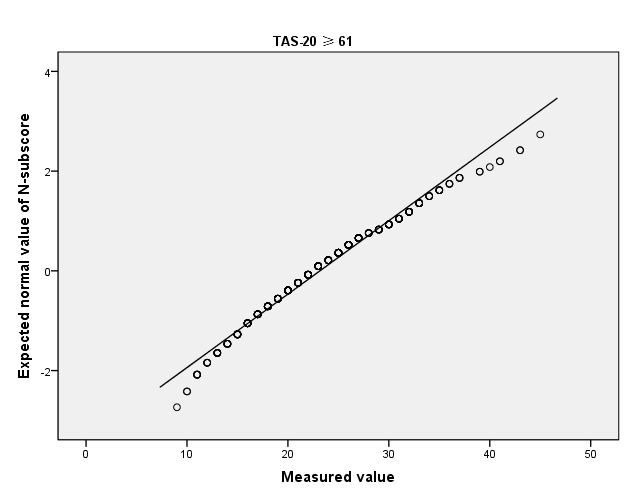


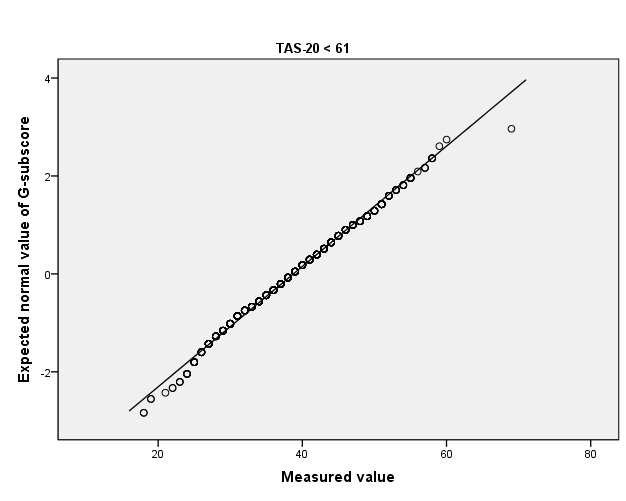

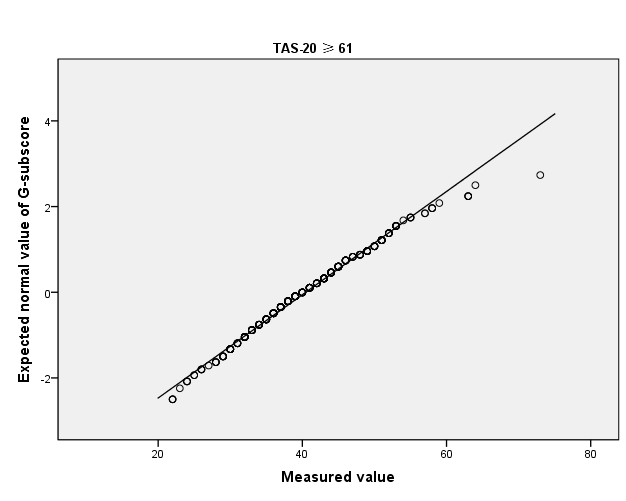


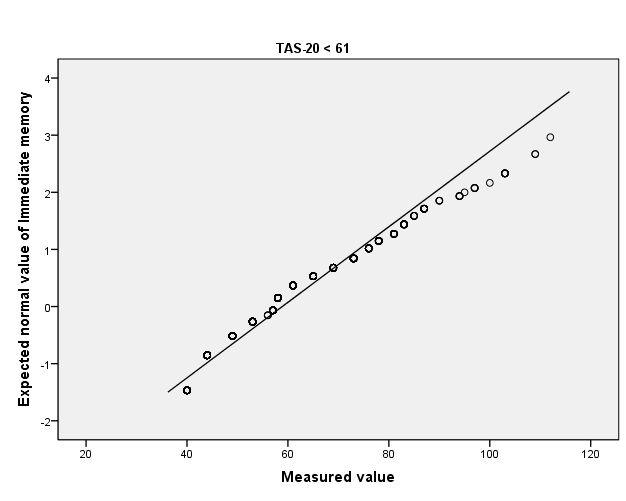

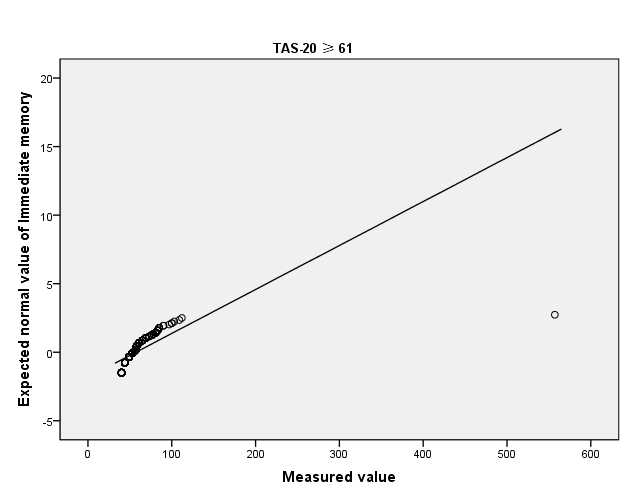

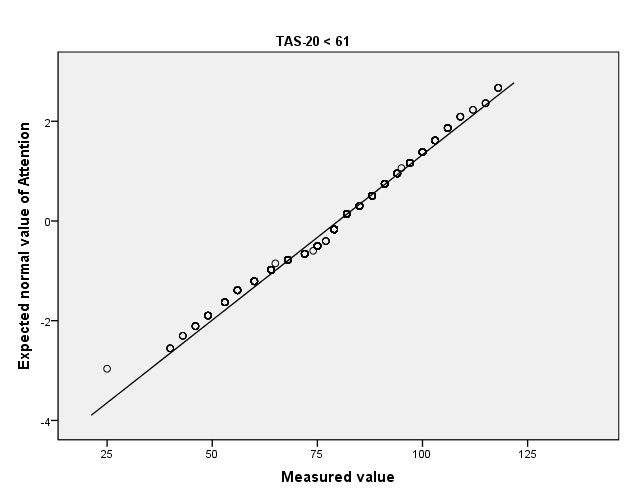

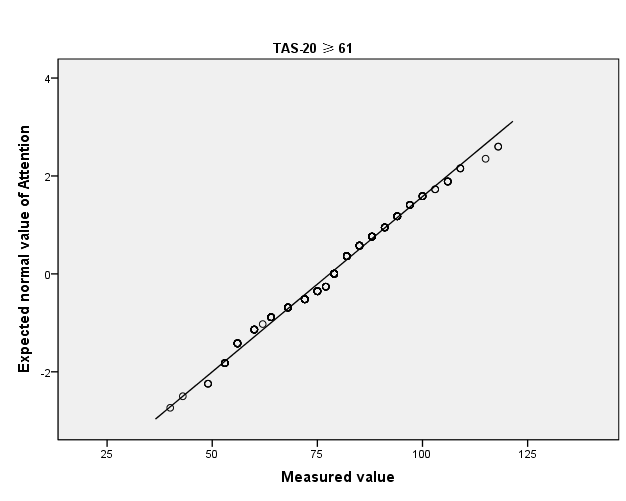


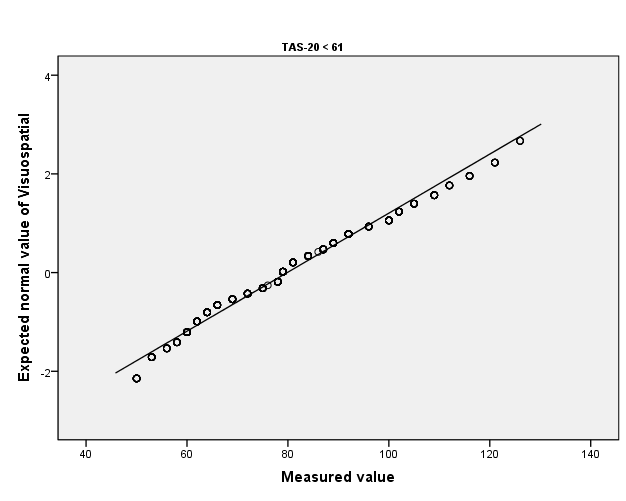

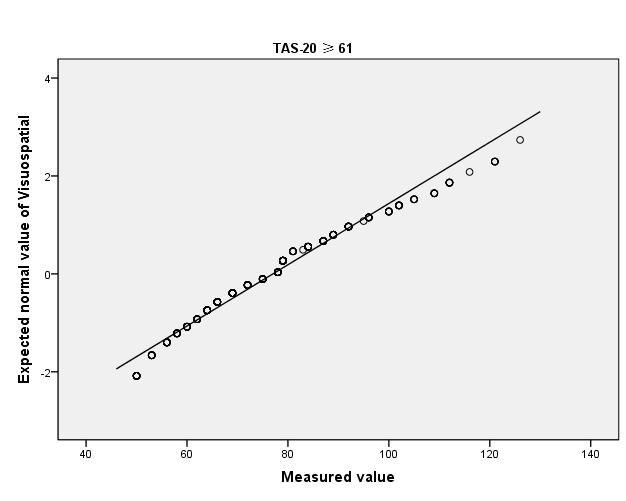

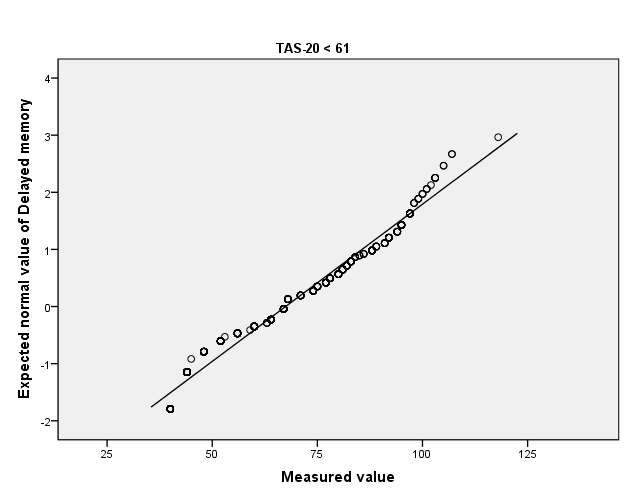

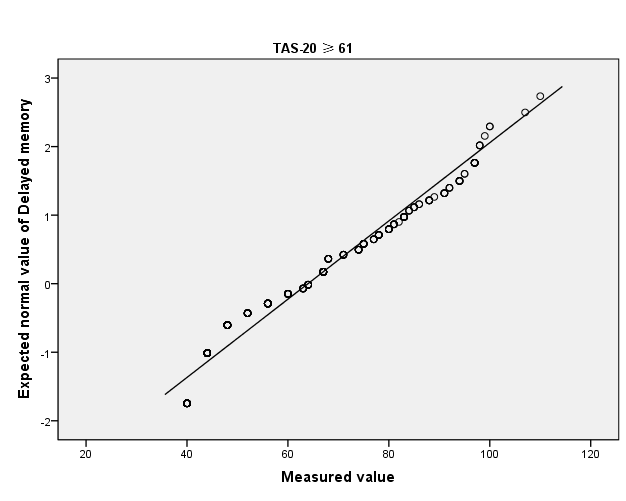


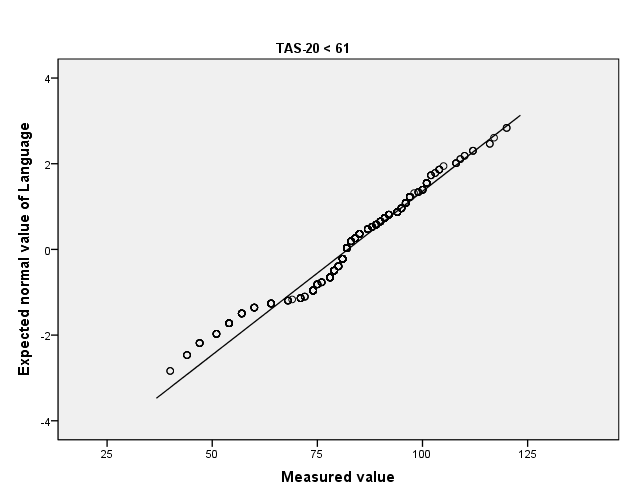

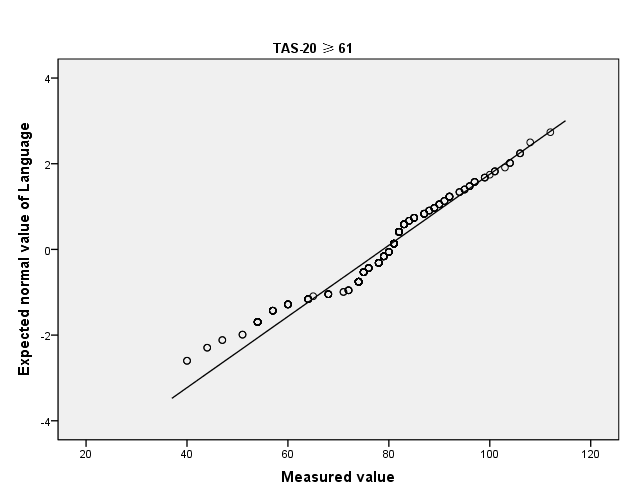


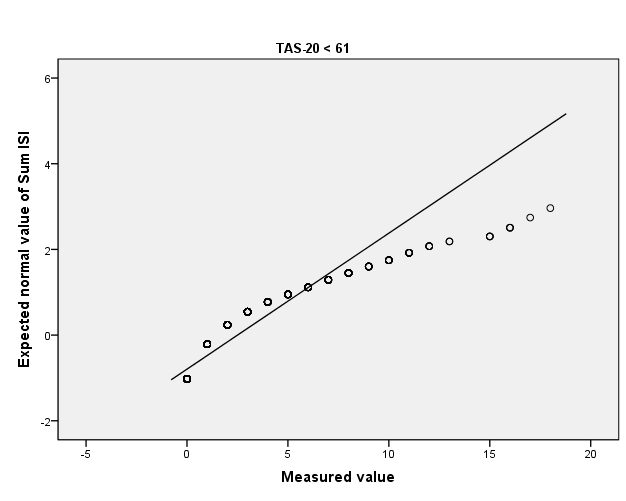

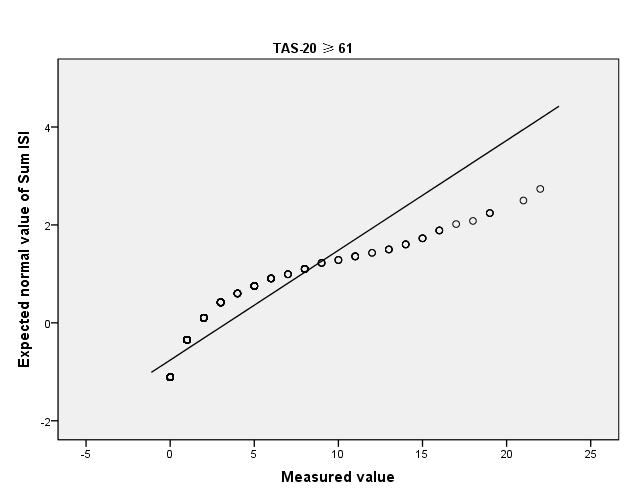


Supplementary Figure2. Mediation effect of RBANS on the link between alexithymia (TAS-DIF) and insomnia in Chinese Patients.

RBANS

-0.060^***^

-0.340^**^

ISI

TAS-DIF

0.199^***^

**Note：**RBANS, Repeatable Battery for the Assessment of Neuropsychological Status; TAS-DIF, Toronto Alexithymia Scale-Difficulties Identifying Feelings; ISI, Insomnia Severity Index. **P< 0.01, ***P< 0.001.

Supplementary Figure 3. Mediation effect of RBANS on the link between alexithymia (TAS-DDF) and insomnia in Chinese Patients.

RBANS

-0.045^***^

-0.340^**^

ISI

TAS-DDF

0.081^*^

**Note：**RBANS, Repeatable Battery for the Assessment of Neuropsychological Status; TAS-DDF, Toronto Alexithymia Scale-Difficulties Describing Feelings; ISI, Insomnia Severity Index. *P< 0.05, **P< 0.01, ***P< 0.001.
